# Biological and environmental determinants in the structuring of ectoparasites in wild carnivores and rodents in central Chihuahua, Mexico

**DOI:** 10.1007/s00436-026-08640-2

**Published:** 2026-02-14

**Authors:** César F. Hernández-Urbina, Roxana Acosta, Jesús A. Fernández, Nathalie S. Hernández-Quiroz, Guadalupe Nelson Aguilar-Palma

**Affiliations:** 1 Departamento de Recursos Naturales, Facultad de Zootecnia y Ecología, Universidad Autónoma de Chihuahua, Periférico Francisco R. Almada Km. 1, Chihuahua, 31453 Chihuahua México; 2 Departamento de Biología Evolutiva, Museo de Zoología “Alfonso L. Herrera”, Facultad de Ciencias, Universidad Nacional Autónoma de México, 04510 Ciudad de México, México

**Keywords:** Chihuahuan desert, Fleas, Grassland, Lice, Ticks, Mammals, Parasites

## Abstract

The study of the structure of ectoparasite communities in wild mammals is fundamental, as it contributes to comprehend the ecological and health dynamics of these systems. This study analyzed the structure of ectoparasite communities in wild carnivores and rodents in central Chihuahua, Mexico, considering biological variables of the hosts (species and sex) and environmental factors such as temperature in one year cycle. A total of 745 ectoparasites were collected, including 738 fleas (seven species, two families), four lice (two species), and one tick species. Seasonal variation and host family significantly influenced the presence and frequency of ectoparasites. Flea species clustered by host group: Pulicidae predominated in carnivores, with *Pulex simulans* being the most abundant species during winter, while Ceratophyllidae dominated in rodents, with *Malaraeus eremicus* as the most abundant flea in autumn-winter and *Jellisonia* sp. nva. in spring-summer. No significant interspecific interactions were observed among flea species. In rodents, no significant relationship was found between the rodent’s sex and the presence of fleas. In northern Mexico, studies on the composition and interactions of ectoparasite communities in wild mammals and their seasonal structuring are scarce. This research provides new records of ectoparasite-host interactions in the state of Chihuahua, Mexico, and updates current knowledge on how biological and climatic factors influence ectoparasite community behavior. These findings offer valuable insights for biology, public health, and species conservation.

## Introduction

Climate change and other environmental issues (deforestation, urbanization, etc.) have altered the composition of wild mammal and parasite communities (Buhler et al. 2021). It has been documented that wild mammals harbor a wide diversity of parasites, including arthropods and helminths. However, the diversity and structure of those communities are affected by the accelerated expansion of human settlements into natural hábitats. This process has incremented the contact between humans and wildlife, thereby facilitating the emergence of zoonosis among them caused by arthropod parasites (Aguirre 2009; Buhler et al. 2021). For this reason, the study of parasite communities in wildlife associated with humans is essential. Within these communities, in recent years, arthropods such as fleas (Insecta: Siphonaptera), lice (Insecta: Phtiraptera), and ticks (Arachnida: Ixodidae) have received growing attention, since multiple species act as vectors of a wide range of infectious pathogens. Having demonstrated the ability to adapt to anthropized environments, these arthropods have become an essential link in pathogen transmission among wildlife, domestic animals, and humans (Hernández-Camacho et al. 2019). Some diseases caused by these pathogens includes rickettsiosis (*Rickettsia rickettsi*, *Rickettsia felis*), bartonellosis (*Bartonella rochalimae*), plage (*Yersinia pestis*), Lyme disease (*Borrelia burgdonferri*), granulocitic anaplasmosis (*Anaplasma phagocytophilum*), and tularemia (*Francicella tularensis*), among others. The spread and emergence of those diseases have a relevant impact on animal and human health (Nieto et al. 2007; López-Pérez et al. 2018), and their occurrence has become increasingly frequent in recent years (Aguirre 2009).

When investigating the ecological and behavioral interactions of parasite communities, wild carnivores and rodents are considered optimal study systems, because of the multiple trophic interactions among them. For example, they act as both predators and preys for a wide variety of species, exhibit strong adaptation to anthropogenic environments, and occupy diverse trophic niches, which are positive correlated with parasite diversity (Owen 1988; Aguirre 2009; Moreno-Rueda and Pizarro 2010; Pulido-Flores et al. 2013). Finally, these mammalian groups have coexisted for millions of years, allowing for long-term host-parasite associations to evolve (Evans et al. 2007). The ectoparasite communities in these mammals are mainly composed of fleas, lice, ticks and mites, and the species present may vary depending on the study area and the season of the year (Bahgat 2013; Crooks et al. 2001). The structure and behavioral patterns of these communities are influenced by host characteristics (species, size, sex, behavior, and diet), interspecific competition with other ectoparasite species, and external factors such as humidity, solar radiation, etc. (Krasnov 2008; Kowalski et al. 2015; López-Pérez et al. 2018; Veitch et al. 2020; Shuai et al. 2022).

Among the various biotic and abiotic factors impacting the ectoparasite communities in wildlife populations, climatic conditions play a key role. Krasnov (2008) noted that most flea species, declines their population numbers during hot seasons like summer, since the majority of larval stages cannot endure temperatures above 33 °C. Shuai et al. (2022) and Peterson et al. (2023), studying the ectoparasite composition in the Daurian ground squirrel (*Spermophilus dairacus*) and small mammals, respectively, also reported a marked decrease in flea abundance during the summer compared with other seasons, while higher activity and abundance of flea species were recorded in spring and autumn.

According to Krasnov (2008), certain host traits such as hair type, skin thickness, and the presence of specific glands can influence ectoparasite diversity. As a result, some parasites may infest specific hosts, giving rise to specificity or generalism. Consequently, differences in ectoparasite richness and abundance may occur even among hosts that share the same ecosystem in time and space. Peterson et al. (2023) emphasized host body size as an essential factor in predisposition to ectoparasite infestation, noting that a larger body surface area provides greater habitat and resources for ectoparasites. This has led to the assumption that in mammals, males show a greater predisposition to infestation than females, and indeed, several studies report significantly higher abundances in males (Kowalski et al. 2015; Veitch et al. 2020; Smith et al. 2021; Shuai et al. 2022; Pontifes et al. 2022; Peterson et al. 2023). The ectoparasite infestation frequencies have also been shown to vary with body size in rodents, where males are generally larger than females (Smith et al. 2021). Furthermore, males are known to experience immunosuppression due to the continuous release of sex hormones, which predisposes them to higher levels of parasite infestation compared with females (Krasnov 2008), suggesting that sex is another key factor influencing the composition and structure of ectoparasite communities in mammals, even within the same species.

In some studies, has been observed potential interactions between different ectoparasite species within the same host (López-Pérez et al. 2018, 2022; Veitch et al. 2020). Within these interactions, negative correlations have been observed between *Pulex simulans* Baker, 1895 and *Echidnophaga gallinacea* Westwood, 1875 (López-Pérez et al. 2018), as well as positive correlations between flea species and fly larvae *Cuterebra* sp., suggesting that the presence of one species of ectoparasite may represent a facilitation or exclusion factor towards other ectoparasite species (Veitch et al. 2020).

Between the host and ectoparasite species that share the same space and time, interactions may have ecological and phyletic explanations. Some flea species may be found in host species that are not their primary hosts, as a result of interactions such as the ectoparasite searching for a suitable host, or because an accidental interaction derived from predation or a change of nest or burrow by the host (Patrick and Harrison 1995; López-Pérez et al. 2018). There are studies that suggest that the relative rates of migration between parasites to other hosts may cause local adaptation processes of the ectoparasite looking for more resources (McCoy et al. 2002). Other studies propose that the specificity of the ectoparasites to a determinate host may disappear as the ectoparasite comes into contact with another host and adapts to other potential host species (Dietrich et al. 2014).

Considering the above, the objectives of this study were: (1) Identify which species of ectoparasites are present and their population proportions in wild carnivores and rodents from Central Chihuahua, Mexico; (2) analyze the diversity and abundance of ectoparasite species in wild carnivores and rodents; (3) compare the structure of ectoparasite community between wild carnivore and rodents; (4) to examine the role of host biological factors (species and sex) and environmental factors (temperature across seasons) in shaping ectoparasite community in wild carnivores and rodents; (5) and to identify potential positive or negative interactions among different ectoparasite species within the same hosts.

## Methods

### Study area and sampling period

This study was conducted in Colonia Soto, Chihuahua, Mexico, located on the Mexican Plateau, a region consisting of broad plains, rolling hills, and small mountain ranges. The area forms part of the Chihuahuan Desert and is characterized by a semi-arid temperate climate with low annual precipitation. Mean annual temperature ranges between 13 and 18 °C, and the study site is situated at an elevation of 1,719 m a.s.l. (N 28°17’50.35”, W 106°06’34.31”). The area corresponds to a micro-basin located within a transitional zone between tufted grassland and oak forest. The warmest months are June and July, with average temperatures of 25–26 °C, whereas the coldest months are December, January, and February, with temperatures around 8 °C. Annual precipitation averages approximately 353 mm, and mean annual relative humidity is around 45% (INEGI 1999; Campos-Trujillo et al. 2015). The most abundant vegetation in the area are *Quercus arizonensis* and *Quercus emoryi*, with lesser representation of *Vachellia farnesiana*, *Prosopis glandulosa*, and *Baccharis salicifolia*, and representative fauna includes carnivores such as puma (*Puma concolor*), bobcat (*Lynx rufus*), black bear (*Ursus americanus*), coyote (*Canis latrans*), gray fox (*Urocyon cinereoargenteus*), racoon (*Procyon lotor*); ungulates such as mule deer (*Odocoileus hemionus*) and white-tailed deer (*Odocoileus virginianus*); rodents such as deer mouse (*Peromyscus* sp.,) Kangaroo rat (*Dipodomys ordii*), and California ground squirrell (*Otospermophilus beecheyi*), as well as several species of migratory birds (Ceballos and Arrollo-Cabrales 2012; Espinoza-Prieto et al. 2015).

### Collection, management and identification of hosts

Medium-sized carnivores were captured using 15 Victor No. 3 rubber-tiped traps, and small carnivores were trapped using five Tomahawk traps, placed every 150 m along 1.5 km transect. Sardine, chicken and liver were used as baits. Rodents were captured using 100 Sherman set in two transects (200 m apart) of 50 traps (10 m apart) using oatmeal mixed with vanilla as bait. Every 72 h, they were moved 1 km apart to prevent recaptures. Traps were checked twice daily (6:00 am and 5:00 pm). Carnivores were chemically immobilized with zolazepam (10 mg/kg for coyotes, 8.8 mg/kg for gray foxes and 8.2 mg/kg for skunks; Larivière and Messier 1996; Larsen and Kreeger 2007), they were muzzled and systematically examined to collect ectoparasites, marking the ventral area with methylene blue to identify recaptures. Rodents were anesthetized with isoflurane in a gas chamber and repeatedly combed from head to tail to collect ectoparasites. After examination, the specimens were released at the trapping site. Handling was supervised by a trained veterinarian and followed the management guidelines of the American Society of Mammologists (Sikes and The Animal Care and Use Committee of the American Society of Mammalogists 2016), the collection of mammals was conducted under SEMARNAT permit No. SGPA/DGVS/05815/22. Carnivores and rodents were identified using taxonomic and photographic keys (Reid 2006; Schmidly and Bradley 2016).

### Identification of ectoparasites

After the host has been brushed and examined, ectoparasites were collected with entomological forceps and preserved in vials with 70% alcohol, labeled with the data of each host. Lice and fleas were mounted on slides following Smith (1958), and identified using taxonomic keys (lice: Tsojanovich and Pratt 1965; fleas: Traub 1950; Hopkins and Rothschild 1953; Stark 1959; Barrera 1958; Rothschild and Traub 1971; Lewis 2000; Acosta et al. 2008; Acosta and Morrone 2003; Hastriter 2004; Salceda-Sánchez 2004). Ticks were identified directly under a stereoscopic microscope using Gregson’s keys (1941). Finally, the specimens were deposited in the Zoology Museum and the Acarology Laboratory of the Faculty of Sciences of the Universidad Nacional Autónoma de México (UNAM).

#### Data analysis

The data of each host were recorded in field sheets (date, coordinates, specie, sex, number of ectoparasites collected), and organized in data matrices in Excel (©2016), including ectoparasite identification. Season (autumn, spring, summer and winter), host species, and sex were used like explanatory variables; response variables included ectoparasite richness, abundance and composition. Seasonal temperature average was obtained from NASA POWER (POWER Prediction of Worldwide Energy Resources); autumn 2022 (15.6 °C); spring 2023 (19.35 °C); summer 2023 (34.6 °C); and winter 2024 (12.62 °C).

#### Sampling coverage and analysis of species diversity

For analyses, the “iNEXT” package from Anne Chao’s Website was used employing Hill numbers to analyze the community assembly of ectoparasites collected from carnivores and rodents, and the sampling effort was evaluated using rarefaction and extrapolation curves with the same package (Chao and Jost 2012). The general formula for Hill numbers was employed according to Hill (1973), Jost (2006), and Ricotta and Feoli (2024): $qD=({\textstyle\sum_{(i=1)}}{\textstyle S}{\textstyle\;}{\textstyle{\scriptstyle p}_i}{\textstyle\;}{\textstyle q})(1/(1-q))$ , where: ^q^D = effective number of species (diversity of order *q*), *pi* = relative abundance of species in the community, *S* = total number of species observed, *q* = diversity order (sensitiviti parameter thet determines the weighting of rare versus common species; Ricotta and Feoli 2024). To describe the structure of the ectoparasite community (richness, abundance and species dominance), the values of *q* when equal to 0, 1 and 2 of each assemblage of ectoparasites obtained were graphed (Hsieh et al. 2016), an exploratory analysis was conducted to assess potential seasonal changes in the structure of the ectoparasite community during autumn (December 2022), spring (April 2023), summer (June 2023), and winter (January 2024), based on the data collected.

#### Comparison of the structure of ectoparasite communities between carnivores and rodents

With the aim of exploring the similarity between ectoparasite communities associated with different mammal hosts during each season, the Jaccard Similarity Index was used in the “vegan” package in R software (Castro-Navarro et al. 2025), taking into account species richness and abundance (Jaccard 1901). The values were transformed to their natural logarithm to linearize the data and reduce bias.

### Ectoparasite-host associations

To analyze the factors that might be influencing the relationship between different species of ectoparasites and their hosts, ectoparasite frequencies were transformed to the natural logarithm. The “Tidyverse” and “Factoextra” packages in R were used, to perform a Principal Component Analysis (PCA; Hotelling 1933) as an exploratory analysis in order to observe the potential relationship between variables (ectoparasite species, hosts and seasons). Only components 1 and 2 were used, as they represent the 70% of the study’s variability.

#### Relative abundance of ectoparasites

To obtain the relative abundance (*pi*), the formula $\:pi\:=\:ni/\varSigma\:ni$, was employed, where: *ni*= number of individualsof species *i*, and Σ*ni*= total number of individuals collected. Subsequently, the base-10 logarithm of (pi) for each species was obtained, which were used to graph the rank-abundance curves for each season and host, wiht the objective of observing abundances in the different species of ectoparasites for each season of the year during the sampling (McGill et al. 2006).

#### Interspecific relationships between ectoparasites

To evaluate the possible interactions between ectoparasite species for host, a Spearman Correlation Analysis in R was carried out (packages “readxl” and “Hmisc”), suitable for data not fitted to the normal distribution (Delucchi 2002). The species with low presence in the study were excluded (lice *Hoplopleura arizonensis* Stojanovich and Pratt 1961, *Linognathus ovillus* Neumann 1907, tick *Ixodes hearlei* Gregson 1941; and flea *Pleochaetis mundus* Jordan and Rotschild 1922) to reduce bias. The Spearman coefficient takes values between 1 and − 1, where the positive values indicates that the abundance of two species tend to increase together, negative values reflect inverse relationships and values equal to 0 indicates absence of relationship.

#### Relationship between the sex of the individual host and the presence of ectoparasites

This analysis included only *Peromyscus attwateri* Allen 1895 and *Peromyscus maniculatus* (Wagner 1845), the species that presented significant abundance of ectoparasites. Due to the low number of individuals of *P. maniculatus*, both species were grouped as *Peromyscus* sp. to reduce bias. A Generalized Lineal Model (GLM) in SAS 9.0 (Allison 2001) with a log link function was used to evaluate the interaction between host sex and ectoparasite presence, using the model: $\:\mathrm{l}\mathrm{o}\mathrm{g}\left({\upmu\:}\right)=\:\mathrm{F}\mathrm{l}\mathrm{e}\mathrm{a}\mathrm{s}\mathrm{Y}\mathrm{e}\mathrm{s}\mathrm{N}\mathrm{o}\mathrm{*}\mathrm{S}\mathrm{e}\mathrm{x}\mathrm{M}\mathrm{F}$, including main effects and their interaction. Where µ represents the expected number of ectoparasites per host, *SexMF* denotes sex of the individual (Male or Female). This analysis could not be applied to carnivores because most of the individuals collected across the three carnivore species were males, therefore this effect could not be observed in those mammals.

#### Relationship between ectoparasites and annual seasons

For this analysis, data were transformed to their natural logarithm. A Generalized Lineal Model (GLM) in SAS 9.0 (Allison 2001) was employed to evaluate possible interactions among ectoparasites and seasons. A model with double interaction (ectoparasite x season) was tested, as well as main effects for each variable. Due to empty spaces at several levels of the model, a Type IV sum of squares was employed. General equation was.

$$\:\mathrm{L}\mathrm{o}\mathrm{g}\mathrm{C}\mathrm{o}\mathrm{u}\mathrm{n}\mathrm{t}=\:\mathrm{S}\mathrm{e}\mathrm{a}\mathrm{A}\mathrm{S}\mathrm{S}\mathrm{u}\mathrm{W}\:+\:\mathrm{E}\mathrm{c}\mathrm{t}\mathrm{P}\mathrm{E}\mathrm{c}\mathrm{h}\mathrm{E}\mathrm{u}\mathrm{M}\mathrm{J}\mathrm{O}\mathrm{P}\mathrm{l}\:+\:\mathrm{S}\mathrm{e}\mathrm{a}\mathrm{A}\mathrm{S}\mathrm{S}\mathrm{W}\mathrm{*}\:\mathrm{E}\mathrm{c}\mathrm{t}\mathrm{P}\mathrm{E}\mathrm{c}\mathrm{h}\mathrm{E}\mathrm{u}\mathrm{M}\mathrm{J}\mathrm{O}\mathrm{P}\mathrm{l},$$

where: Sea= Season, Ect= Ectoparasite, and the levels are: A= Autumn, S=Spring, Su=Summer, W= Winter, P= *Pulex*, Ech= *Echidnopaga*, Eu= *Euhoplopsyllus*, M= *Malaraeus*, J= *Jellisonia*, O= *Orchopeas* and Pl= *Pleochaetis*. However, interactions analysis could not apply due to low presence and, multiple cells with zero values, which prevented obtaining sufficient degrees of freedom for statistically significant results. Therefore, only the principal effects were considerate with significant level of 95%.

## Results

Total of 26 carnivores grouped in two families, *Canidae* (*Canis latrans* Say 1823 and *Urocyon cinereoargenteus* Schreber 1775) and *Mephitidae* (*Mephitis mephitis* Schreber 1776), and three species were captured, being the last species captured in all seasons, while *C. latrans* and *U. cinereoargenteus* were not captured in at least one season of the year (Table 1). Regarding rodents, 250 individuals were collected from nine species in two families: Cricetidae (five species) and Heteromyidae (four species). The rodent species with the highest number of individuals in all seasons were *Peromyscus attwateri* and *P. maniculatus*, while *Dipodomys ordii*, *Sigmodon hispidus*, *Chaetodipus hispidus*,* Chaetodipus* sp. and *Microtus mexicanus* had low numbers (Table 1).

**Table 1:** Total number of specimens per species captured by season of the year during field sampling

Species	Autumn	Spring	Summer	Winter	Total
Carnivora					
*C. latrans*	3	3	1	-	7
*U. cinereoargenteus*	3	1	-	7	11
*M. mephitis*	1	4	1	2	8
Rodentia					
*P. attwateri*	57	48	45	14	164
*P. maniculatus*	12	20	13	3	48
*P. leucopus*	-	-	14	-	14
*S. hispidus*	-	-	2	-	2
*Ch. hispidus*	-	-	1	-	1
*Chaetodipus* sp.	1	-	1	-	2
*D. ordii*	1	-	-	3	4
*P. flavus*	3	3	8	-	14
*M. mexicanus*	1	-	-	-	1

The ectoparasites collected in carnivores and in four of the nine rodent species (*P. attwateri*, *P. maniculatus*, *Peromyscus leucopus* and *S. hispidus*) (Table 2), correspond to ten species, classified in three orders: Siphonaptera (two families and seven species), Phthiraptera (two families and two species), and Arachnida (one family and one species) (Table 2). However, carnivore and rodent communities share only one species of flea *M. eremicus* Baker 1904. Flea species *Pulex simulans* and *Echidnophaga gallinacea* were more frequently collected in *M. mephitis* and *U. cinereoargenteus*, whereas they were less frequently collected from *C. latrans* they were. On the other hand, there were two species of ectoparasites that were scarce, the tick *Ixodes hearlei* in *M. memphitis* and the louse *Linognathus ovillus* in *U. cinereoargenteus*. Among rodents, the flea species *Jellisonia* sp. nva. and *M. eremicus* were represented by a higher number of specimens in *P. attwateri* and *P. maniculatus*, in contrast *Orchopeas leucopus* Baker 1904 was registered in three rodent species in low numbers. Finally *Pleochaetis mundus* and the louse *Hoplopleura arizonensis* were each found in a single species (*P. attwateri* and *S. hispidus* respectively) (Table 2).

**Table 2:** Family, species, number, and percentage of ectoparasites recorded in infested carnivores and rodents throughout the sampling period. Each cell shows the number of ectoparasites present/percentage of infested hosts

Ectoparasite/host (total number)	C. latrans (7)	U. cinereoargenteus (11)	M. mephitis (8)	S. hispidus (2)	*P*. attwateri (164)	*P*. maniculatus (48)	*P*. leucopus (14)
Pulicidae							
*P. simulans*	66/100	98/90.9	248/100	-	-	-	-
*E. gallinacea*	3/42.85	10/27.3	37/37.5	-	-	-	-
*E. glacialis affinis*	1/14.3	5/27.3	-	-	-	-	-
Ceratophyllidae							
*Jellisonia* sp. nva.	-	-	-	-	68/18.3	5/4.2	-
*M. eremicus*	1/14.3	-	-	-	142/26.2	35/18.8	-
*O. leucopus*	-	-	-	-	15/4.3	7/6.3	1/7.1
*P. mundus*	-	-	-	-	4/1.2	-	-
Hoplopleuridae							
*H. arizonensis*	-	-	-	3/50	-	-	-
Linognathidae							
*L. ovillus*	-	1/9	-	-	-	-	-
Ixodidae							
*I. hearlei*	-	-	3/25	-	-	-	-

### Sampling effort and species diversity

**Fig. 1:**
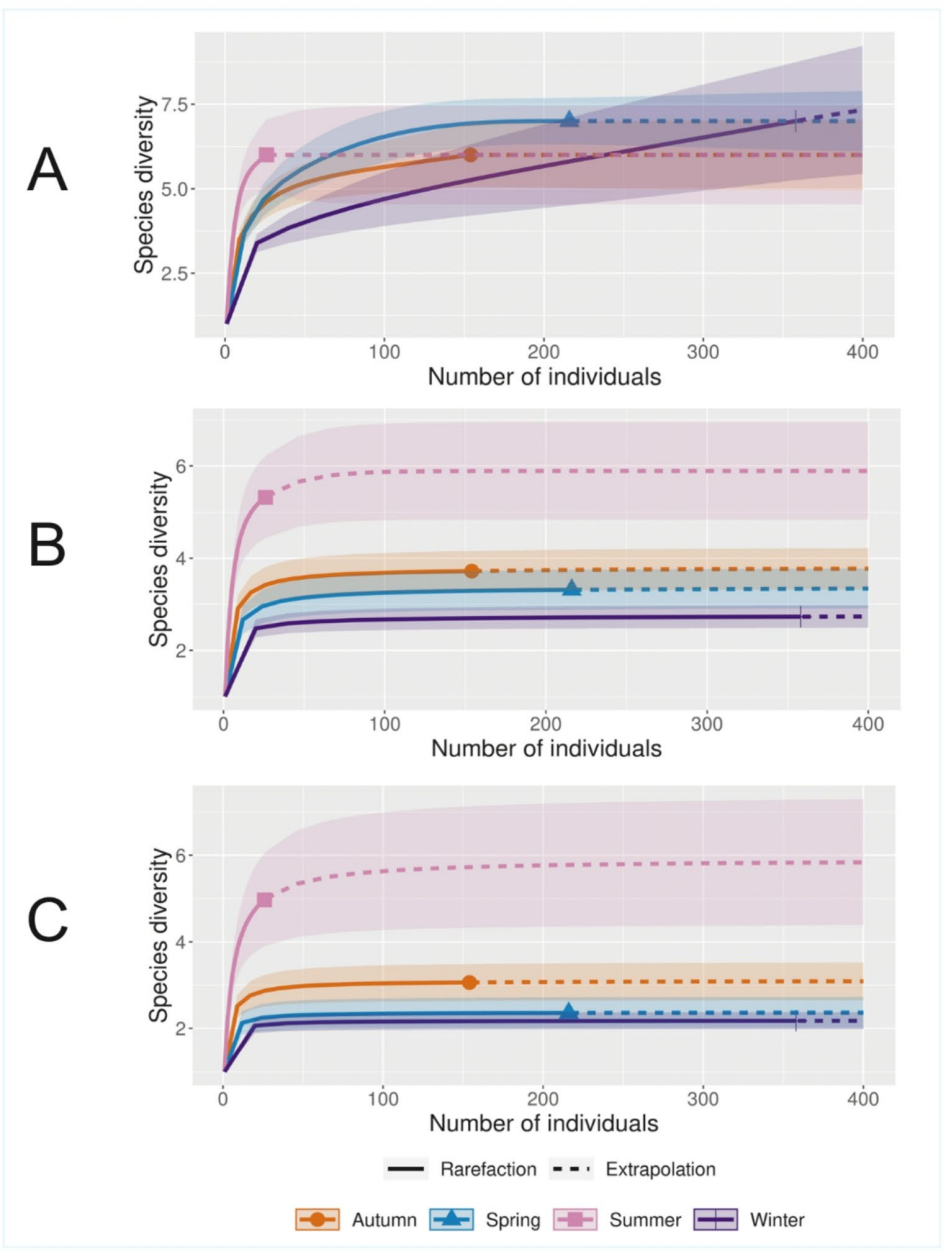
Rarefaction curves showing (**A**) species richness (q = 0), (**B**) diversity of common species (q = 1), and (**C**) species dominance (q = 2) of ectoparasites including carnivores and rodents by season of the year

The sampling effort for each season exceeded the minimum required (90%), reaching the asymptote and taking into account the data extrapolation. The analysis of species richness (q = 0) in carnivores and rodents showed no clear differences among seasons, with a similar number of species recorded across sampling periods. For common species (q = 1), summer exhibited higher ectoparasite diversity compared to the other seasons, as suggested by the rarefaction and extrapolation curves and their confidence intervals. No marked differences were observed among autumn, spring, and winter. In the analysis of dominant species (q = 2), summer also showed higher diversity values, indicating the presence of dominant ectoparasite species during this season. Winter was the only season in which species richness (q = 0) does not reach an asymptote (Fig. 1).

### Comparison of the composition of ectoparasite communities between carnivores and rodents

The Jaccard Analysis identify two ectoparasite groups and revealed low similarity between carnivores and rodents ectoparasite communities, with only one shared species *C. latrans* and rodents (14% of similarity). Fleas parasitizing Carnivore and rodent belonged primarily to the families Pulicidae and Ceratophyllidae respectively (Table 2). *Canis latrans* and *U. cinereoargenteus* showed high similarity of 75%, whereas *M. mephitis* exhibited low similarity with the canids. Among rodents, *P. attwateri* and *P. maniculatus* showed great similarity (75%) to each other than to the remainig seven rodent species (Table 3).

**Table 3:**
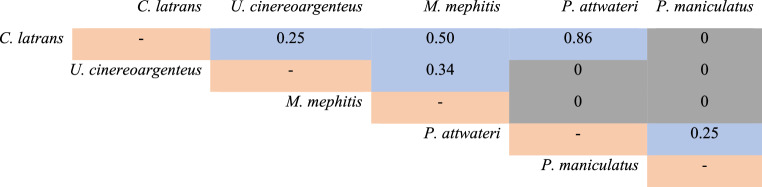
Similarity values based on ectoparasite presence among different hosts using Jaccard Similarity Analysis. Blue cells indicate some degree of similarity, gray cells represent no similarity, and brown cells correspond to comparisons of the same individual

### Ectoparasite-host

**Fig. 2:**
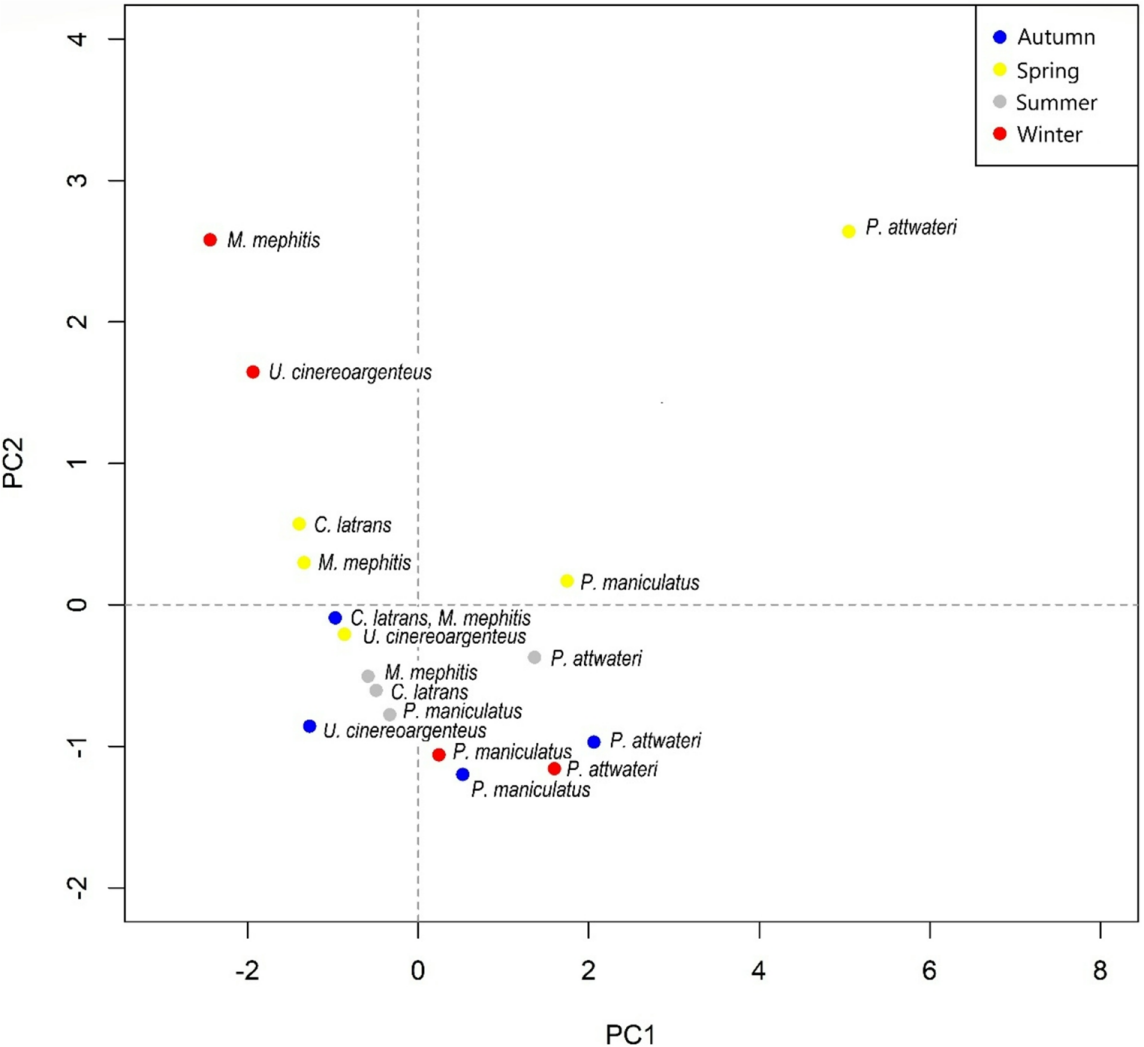
Principal component analysis (PCA) plot of ectoparasite communities in carnivores and rodents. Carnivores cluster on the left side, while rodent group on the right, reflecting distinct ectoparasite communities

The Principal Component Analysis (PCA) shows that principal components 1 and 2 with a variance proportion of 0.47 and 0.20 respectively, explain almost 70% of the data variability (0.67). PCA suggests differences in the frequencies throughout the seasons of the year. This analysis also shows a clear segregation of the ectoparasite communities, one group associated with carnivores and the other with rodents, like the previous analyses (Fig. 2).

### Relationship between the sex of the individuals and the presence of ectoparasites

The results of the General Lineal Model show that the interaction between host sex variable and the presence of ectoparasites in rodents are marginally non-significant (confidence level of 95%; *p* = 0.0542; Table 4). However, on average, males show a higher number of infested individuals than females (134 males with 51 fleas and 75 females with 28 fleas; Table 5).

**Table 4:** List of generalized lineal models employed during this study with their respective coefficients, factors and p-values

Model	Variation coefficient	Factors	F values	*P* values
log(µ)= FleasYesNo*SexMF	194.94	Fleas	90.44	< 0.0001
		Sex	3.75	0.0542
		Fleas * Sex	3.75	0.0542
LogCount= SeaASSuW + EctPEchEuMJOPl +SeaASSW* EctPEchEuMJOPl	51.15	Season	3.64	0.0327
		Ectoparasites	8.16	0.0002
		Season * Ectoparasites	1.86	0.1102

**Table 5:** Overall flea infestation percentages by sex in *Peromyscus* rodents across seasons during the entire sampling period

Rodents	Sex	Autumn	Spring	Summer	Winter	Total
*Peromyscus* sp.	Male	47.7%	40%	13.6%	80%	38.5%
	Female	40%	38%	16.6%	85%	36.4%

### Relationship between ectoparasites and annual season

According with the significant values obtained in the Generalized Lineal Model (GLM), two variables show a statistically significant effect in the model: variable “season” (*p* = 0.0327), and “ectoparasite” variable (*p* = 0.0002). This indicates that the ectoparasite abundances vary significantly as the seasons change within the GLM model.

### Temporal variation of ectoparasites in carnivores and rodents

**Fig. 3:**
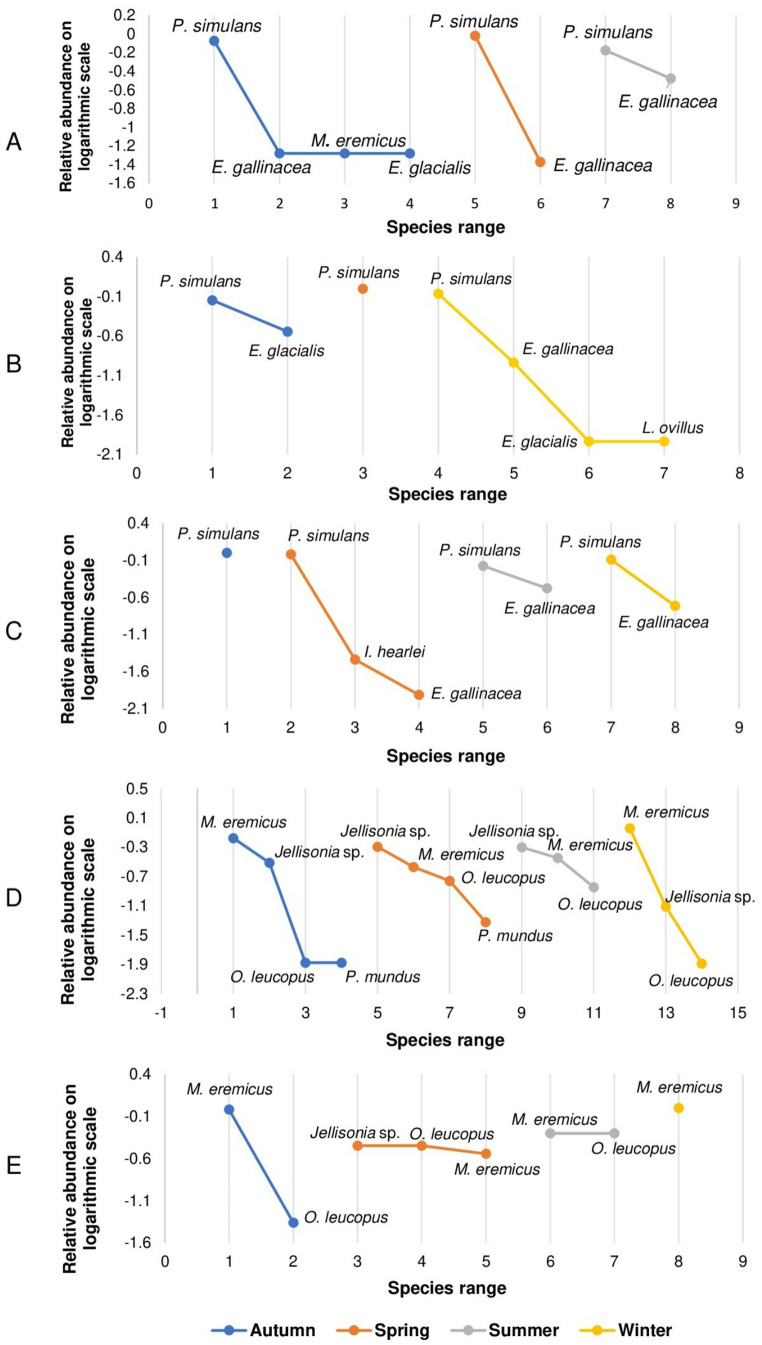
Rank-abundance curves of ectoparasite species recorded by season in (**A**) *Canis latrans*, (**B**) *Urocyon cinereoargenteus*, (**C**) *Mephitis mephitis*, (**D**) *Peromyscus attwateri*, and (**E**) *Peromyscus maniculatus* throughout the sampling period

The relative abundance analysis shows seasonal variations in the ectoparasite community of carnivores (Fig. 3). *Pulex simulans* was the most dominant on the three carnivore species, with proportions greater than 60% in all seasons, reaching its peak in spring (100% *U. cinereoargenteus*, 96% *C. latrans* and 95% *M. mephitis*) although *E. gallinacea* is present in low numbers, the winter period was characterised by an increase in this flea species on *M. mephitis* (19%). Three ectoparasite species with low presence were registered: *Euhoplopsyllus glacialis affinis* and *M. eremicus* in spring and autumn on *C. latrans* and *U. cinereoargenteus*, and *Ixodes hearlei* on *M. mephitis* only on spring. In rodents, flea dominance has variation according to the season. *Malaraeus eremicus* predominated in autumn and winter on *P. attwateri* and *P. maniculatus* (67% and 96%, respectively), decreasing on spring and summer. On the contrary, *Jellisonia* sp. nva. was more abundant in warm seasons, in spring (51%) and summer (50%). While *O. leucopus* shows an apparent seasonality, with higher proportions in spring and summer, although with low proportion. *Pleochaetis mundus* was scarce during all seasons, with no clear evidence of seasonality in this species (Table 2; Fig. 3).

### Interspecific relationships between flea species

The Spearman Correlation Analysis suggests positive and negative interspecific interactions among some flea species in carnivores and rodents. Positive correlations were detected (+ 0.8) between *P. simulans* and *E. gallinacea* in carnivores, and between *O. leucopus* and *Jellisonia* sp. nva. (+ 0.8) in rodents. It was also detected a possible negative interaction (−0.8) between *M. eremicus* and *O. leucopus* in rodents. However, despite the high correlation values, the *p* values are not statistically significant in none of the presented interactions (Tables 6 and 7).

**Table 6:**
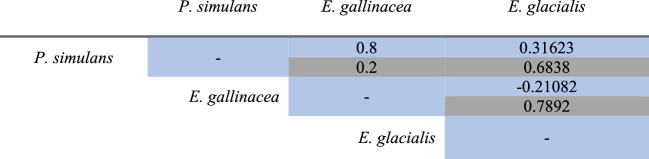
Spearman correlation values obtained for flea species collected from carnivores (*C. latrans*, *U. cinereoargenteus*, and *M. mephitis*). Values in blue represent the correlation coefficients, while those in gray correspond to the p-values

## Discussion

The ectoparasite community in wild carnivores and rodents were composed of ten species. These included seven fleas (Siphonaptera), two lice (Phthiraptera) and one tick (Ixodida). Fleas represented more than 50% of the registered species in these wild mammals. This pattern is consistent with previous studies conducted in North America, the United States of America (USA), Mexico, and Europe, particularly in temperate and arid zones (Brinkerhoff 2008; Gabriel et al. 2009; Sréter et al. 2003; Crooks et al. 2001, 2004). This structuring pattern of ectoparasites was registered in *Vulpes vulpes* in Hungria and Spain (Sréter et al. 2003), *Vulpes velox* in USA (Brinkerhoff 2008; Gabriel et al. 2009) and *Urocyon litoralis* and *Spilogale gracilis amphiala* in Texas (Crooks et al. 2001, 2004). As for rodents, Islam et al. (2021), who studied 87 species in the Middle East and López-Pérez et al. (2022) with five species in Chihuahua, México, reports that the collected ectoparasites were also fleas. These proportions may be explained by the fact that flea species are generally considered to be moderately generalists, as they possess evolutionary traits that have enabled them to adapt to multiple ecosystems and to parasitize a broad diversity of vertebrates worldwide. Although fleas spend much of their life cycle on the host, they are capable of moving freely among hosts, facilitating access to new hosts; consequently, their distribution is closely related with host range (Krasnov 2008; Krasnov and Khokhlova 2001; Marshall 1981; Kuznetzov et al. 1999). Other ectoparasites such as ticks who presents a restricted movement, depending almost exclusively on specific microhabitats or of special characteristics of local vegetation to locate themselves and passively wait for a potential host (Schulz et al. 2014).

**Table 7:**
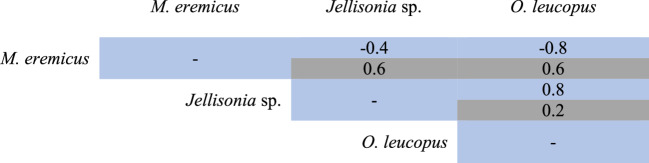
Spearman correlation values obtained for flea species collected from rodents (*P. attwateri* and *P. maniculatus*). Values in blue represent the correlation coefficients, while those in gray correspond to the p-values

The flea community in the three carnivores studied, was composed of *E. gallinacea*, *P. simulans*, and *E. glacialis affinis* (Acosta 2005; Gyimesi et al. 2007; López-Pérez et al. 2018), these is consistent with other studies (Hernández-Urbina et al. 2025). The genus *Pulex* is generally associated with wild carnivores such as *U. cinereoargenteus* (Gabriel et al. 2009), C. *latrans* (López-Pérez et al. 2018), V. *macrotis* (Harrison et al. 2003), V. *velox* (Salkeld et al. 2007), U. *littoralis* (Crooks et al. 2001), S. *gracilis amphiala* (Crooks et al. 2004), M. *mephitis* (Brinkerhoff 2008), and *V. vulpes* (Sréter et al. 2003). In Chihuahua, Mexico, *P. irritans* and *P. simulans* have been recorded in wild carnivores, with the former mainly associated with *V. macrotis*, while *P. simulans* is more frequently found in *C. latrans* and *U. cinereoargenteus* (López-Pérez et al. 2018; Hernández-Urbina et al. 2020). In this study, *P. simulans* was the most abundant ectoparasite species among the three sampled carnivore species, whereas *P. irritans* was not found, consistent with previous reports in the state of Chihuahua. For instance, López-Pérez et al. (2018) documented the presence of *P. irritans* in *C. latrans* in the Janos Biosphere Reserve in northwestern Chihuahua, although its abundance was very low. Similarly, Hernández-Urbina et al. (2020) reported *P. irritans* as the only species present in coyotes in the northern region of Samalayuca, Juárez. These findings may support the hypothesis of a latitudinally differentiated distribution of *Pulex* species and their hosts, as suggested by Hopla (1980) in López-Pérez et al. (2018), since *P. irritans* appears to be less abundant or even absent in the southern portion of the coyote distribution range, where *P. simulans* shows higher prevalence. *Echidnophaga gallinacea*, although typically associated with birds, also parasitizes mammals (Falcón-Ordaz et al. 2012; Frixione et al. 2023). This species was the second most abundant, parasitizing *M. mephitis*. Mead (1963) and Greenwood et al. (1999) suggested that this association may be related to the skunk’s habit of raiding bird nests. In contrast, *C. latrans* and *U. cinereoargenteus* showed low infestation rates (Eads 1948; Turkowski 1974; Patrick and Harrison 1995). However, this flea is more strongly associated with *Lynx rufus*, *Procyon lotor*, and *Taxidea taxus*, where higher abundances have been reported (Layne 1971; López-Pérez et al. 2018). The presence of *E. glacialis affinis* in *C. latrans* and *U. cinereoargenteus* may be attributable to accidental infestations, as this flea typically parasitizes their prey (rabbits and hares; López-Pérez et al. 2018; Hernández-Camacho et al. 2019; Patrick and Harrison 1995).

In the rodents sampled, the flea community comprised four species: *Malaereus eremicus* and *Jellisonia* sp. nva. are common in desert and semi-arid habitats of North America and were the most abundant species observed in this study, primarily parasitizing rodents of the genus *Peromyscus*, although they can also infest other species (Beer et al. 1959; Traub et al. 1983a, b; Ayala-Barajas et al. 1988; Hastriter and Eckerlin 2003; Hastriter 2004; Acosta 2005; Acosta et al. 2008; Lewis 2008; Falcón-Ordaz et al. 2012; Zapata-Valdés et al. 2020; Peterson et al. 2023). Conversely, *O. leucopus* and *P. mundus* are typically associatedwith montane and temperate habitats (Dampf 1942; Verts 1961; Traub et al. 1983a, b; Lewis 2008), which may account for their low abundance in the study area, where environmental conditions differ from those of their optimal habitat.

Diversity analyses did not reveal significant differences in species richness (q = 0) of ectoparasites between carnivores and rodents throughout the year. This uniformity may be attributed to the presence of generalist or widely distributed species across the continent, which exhibit high climatic tolerance (López-Pérez et al. 2018; Lewis 2008; Eckerlin 2016; Hastriter and Eckerlin 2003), and are therefore able to persist across all seasons. It has been documented (Peterson et al. 2023; Morlan 1955) that some of these species remain active year-round, though with fluctuations in abundance, consistent with the patterns observed here. During winter, the rarefaction curve did not reach an asymptote, indicating potencial undersampling and a likely underestimated of species richness for this season, therebyin highlighting the need for increase in sampling effort. Regarding effective diversity (q = 1) and dominance (q = 2), indicated that summer exhibited the highest species presence and the greatest evenness in abundance distribution. This pattern may be explained by an overall reduction in ectoparasite abundance during this season, possibly associated with high temperatures, that may constrain life cycle development or reproductive success in some species (Krasnov 2008). Thus, although the number of species remained stable, their relative abundances were more homogeneous. According to Peterson et al. (2023), M. *eremicus*, *O. leucopus*, and *Pleochaetis* sp. reduce their abundance in summer, persisting only in minimal proportions. A similar trend was observed in this study with these species, perhaps contributing to a more even community structure during summer. This pattern reflects how extreme environmental factors could reduce dominance and promote a more balanced distribution among species.

The ectoparasite communities associated with wild carnivores and rodents differ markedly, sharing only one species: *M. eremicus* in one specimen of *C. latrans*, and is considered an accidental infestation (Lewis 2000; López-Pérez et al. 2018; Hernández-Urbina et al. 2025). This structure suggests that the specific composition of ectoparasites is strongly influenced by the host identity. In the carnivores, *C. latrans* and *U. cinereoargenteus* shared three species of collected fleas (*P. simulans*, *E. gallinacean*, and *E. glacialis affinis*), and *M. mephitis* shared two species (*P. irritans* and *E. gallinacea*) with the other carnivore species. This pattern may be explained by the widespread occurrence and high prevalence of *Pulex* fleas in wild canids and mephitids across other regions of the world, while *E. gallinacea* is likewise a generalist species that has been frequently recorded in these wild carnivores (Eads 1948; López-Pérez et al. 2018). In rodents, *P. attwateri* and *P. maniculatus* share three flea species that are common in rodent species of the genus *Peromyscus*. This affinity reflects established patterns of parasite-host in other regions of Mexico (Acosta 2005; Lewis 2008; Falcón-Ordaz et al. 2012).

Seasonality can significantly influence the dynamics of certain ectoparasites species, as environmental factors such as solar radiation and relative humidity directly affect the immature stages and the reproductive cycle of fleas (Krasnov 2008; Pontifes et al. 2022; Shuai et al. 2022). In this study, seasonal variations were observed in the community structure of ectoparasites associated with wild carnivores and rodents along the year, likely in response to changes in climatic conditions such as temperature. Ectoparasite abundance decreased considerably in summer on wild carnivores and rodents, while in winter the ectoparasite abundance increases for some of the flea species. In Janos Biosphere Reserve, Chihuahua, Mexico, López-Pérez et al. (2018) reported that *P. irritans* and *E. gallinacea* are more strongly associated with wet seasons. In contrast, Crooks et al. (2001), in a study *U. littoralis* in Santa Cruz Island, California, found that mites and ticks (*Neotrichodectes mephitidis* and *Ixodes pacificus*) exhibit higher prevalence during these seasons, suggesting that wet conditions provide optimal contitions for life cycles.

In rodents, Peterson et al. (2023) documented seasonal variations in flea species composition in New Mexico: *Aetheca wagnery* was more frequent in spring, while *Pleochaetis exilis* predominated in autumn. During summer, a significant reduction in flea richness and abundance was reported, as also observed in our study; however, *O. leucopus* remained relatively more abundant during this season. In the present study, an apparent seasonality was also observed between *Jellisonia* sp. nva. and *M. eremicus*, the two most prevalent species in *P. attwateri* and *P. maniculatus*. *Jellisonia* sp. nva. was more frequent in warm seasons, while *M. eremicus* predominated in colder ones. According to Morales (1990), Hastriter (2004), and Acosta-Gutiérrez (2014), some *Jellisonia* species are distributed in arid and warm regions, characteristics found in our study area. Nevertheless, *Jellisonia* sp. nva. has been recorded only in the Mexican Plateau (Chihuahua and Durango) (pers. comm. A.G.D. and R.A.G.). In carnivores, despite detectable fluctuations in ectoparasite abundance, the overall community structure remained stable throughout the year, with *P. simulans* as the dominant species, followed by *E. gallinacea*. All flea species exhibit reduced abundance during summer when temperatures exceed 33 °C, consistent with the findings Krasnov (2008), who reported that most fleas do not tolerate temperatures above this threshold due to egg and larval desiccation. The same pattern was reported by Peterson et al. (2023) in rodents from New Mexico, USA. Similarly, Haiwen et al. (2024) documented a significant decline in flea abundance in rodents from Mongolia and China during summer, with increases in autumn and spring. Durden et al. (2005) observed that *P. simulans* reached its highest abundance on domestic dogs during August in Georgia, USA, coinciding with the onset of the rainy season, when residual humidity may favor flea persistence into winter. Adequate humidity levels enhance the survival of all developmental stages, promote normal larval activity, stimulate proper silk production resulting in well-formed and firm cocoons, and support optimal adult emergence and body size (Krasnov et al. 2001; Marshall 1981). In contrast, winter appeared to favor *P. simulans*, *E. gallinacea*, and *M. eremicus*. This pattern has been described in some ectoparasites capable of surviving low temperatures by remaining within the microclimate generated by the host’s fur (Krasnov 2008). This survival strategy may explain the higher winter abundance of *P. simulans* and *E. gallinacea* in carnivores and *M. eremicus* in rodents, observed in this study. Regarding host sex as a factor influencing ectoparasite infestation, no significant differences were observed in infestation levels between males and females. However, a slight increase in flea presence was detected in male rodents of the genus *Peromyscus* coinciding with some authors (Peterson et al. 2023; Veitch et al. 2020; Haiwen et al. 2024). Krasnov (2008) suggested that continuous androgen secretion can induce a degree of immunosuppression, thereby increasing susceptibility of males to ectoparasite infestation. Likewise, larger body size represents a greater surface area that provides a variety of niches and resources for ectoparasites (Kowalski et al. 2015; Veitch et al. 2020; Smith et al. 2021; Shuai et al. 2022).

Positive interactions were identified between some species of ectoparasites, highlighting an apparent association observed between *P. simulans* and *E. gallinacea* (correlation index = + 0.8) in carnivores; and between *M. eremicus* and *O. leucopus* (−0.8) in rodents. Although these associations did not result statistically significant (*p* = 0.2), it was possibly due to the limited sample size for both mammal groups. The pattern observed in the carnivores from this study, contrast with López-Pérez et al. (2018, 2022), who documented exclusion in the Janos Biosphere Reserve, Chihuahua, Mexico, between *P. simulans*, *E. gallinacea* and *P. irritans*, and in Mojave, California, López-Pérez et al. (2022) reported positive associations between flea species and fly *Cuterebra* sp. larvae in *P. maniculatus*. Although the mechanisms governing these interactions between ectoparasites are not fully understood, it has been proposed that they may include direct competition such as physical contact in the host, or indirect competition, through modification of the microenvironment or host resources (blood). These interactions could vary temporally and spatially, and increase sampling effort may be necessary to evaluate the presence of exclusion or facilitation processes among ectoparasite species in these wild mammals.

Herein, we emphasized the importance of understanding the distribution, structuring patterns, and dynamics of ectoparasite communities in wild mammals. Such knowledge is relevant not only from an ecological perspective but also for its implications in other disciplines, including public health and biodiversity conservation. The flea species *P. irritans*, *E. gallinacea* and *O. leucopus* reported in this study are recognized vectors of pathogens of medical importance, such as *Yersinia pestis*, *Bartonella* and *Rickettsia* (Reeves et al. 2005; Fernández-González et al. 2016; Peniche-Lara et al. 2015). This underscores the zoonotic risk in areas where wildlife, domestic animals and human settlements converge, particularly in urban and peri-urban areas of Northern Mexico (García-Rejon et al. 2021). This risk is further exacerbated by climate change and the landscape transformation, which alter the distribution of hosts and their ectoparasites. Although ectoparasites are part of biodiversity, they may represent a threat to vulnerable wildlife under environmental stress or anthropogenic pressure. The identification of novel host-parasite associations (*U. cinereoargenteus*–*Linognathus ovillus* [louse], *Sigmodon hispidus*–*Hoplopleura arizonensis* [louse]) and the discovery of undescribed species (*Jellisonia* sp. nva.) highlight the urgent need for systematic monitoring of parasite biodiversity in Northern Mexico (Hernández-Urbina et al. 2025; Sánchez-Montes et al. 2013).

## Conclusion

This study provides evidence that wild carnivores and rodents host differentiated ectoparasite communities structured by both host biological traits and seasonal environmental variables in central Chihuahua, Mexico. The ectoparasites with higher prevalence were fleas (Siphonaptera), family Pulicidae in carnivores and Ceratophyllidae in rodents. Additionally, we report new host-parasite associations and a previously undescribed species (*Jellisonia* sp. nva), highlighting the need to further explore the poorly studied areas of Northern Mexico. Seasons have influence in the parasite community compositions varied across seasons, with a pattern of greater evenness in summer. Seasonal responses differed between carnivores and rodents, suggesting distinct ecological and thermal survival strategies. The marked differences in ectoparasite communities between these mammalian groups indicate the presence of ecological barriers that may limit cross-transmission. Furthermore, potential negative and positive interactions among ectoparasites were identified, reflecting complex dynamics of coexistence, competition, or facilitation, processes that requires further sampling and integration of ecological variables to be fully understood. These findings have direct implications for public health and biodiversity conservation, as several flea and tick species detected in this study are recognized vectors of rickettsial pathogens. In recent decades, climate change and land-use change have altered the distributional ranges of these ectoparasites, resulting in modified spatial patterns. Our results contribute to the monitoring of ectoparasite communities by providing valuable information for epidemiological studies, particularly in light of emerging risks at the wildlife–domestic animal–human interface. Furthermore, they highlight the urgent need to establish programs that integrate biodiversity conservation strategies with the prevention of emerging infectious diseases.

## Data Availability

Data are available from the authors upon reasonable request.

## Declarations

The collection and sampling of the specimens recorded in this study were conducted under collection permit SGPA/DGVS/05815/22. Capture, handling, and restraint techniques followed the guidelines established by the American Society of Mammologists.
